# Associations of cerebrovascular disease and Alzheimer’s disease pathology with cognitive decline: Analysis of the National Alzheimer’s Coordinating Center Uniform Data Set

**DOI:** 10.1016/j.neurobiolaging.2024.06.002

**Published:** 2024-06-21

**Authors:** Ankita Chatterjee, Shannon Lee, Valentina Diaz, Rowan Saloner, Mark Sanderson-Cimino, Charles deCarli, Pauline Maillard, Jason Hinman, Keith Vossel, Kaitlin B. Casaletto, Adam M. Staffaroni, Emily W. Paolillo, Joel H. Kramer

**Affiliations:** a Memory and Aging Center, Department of Neurology, Weill Institute for Neurosciences, University of California, San Francisco, USA; b Department of Neurology, University of California, Davis, USA; c Mary S. Easton Center for Alzheimer’s Research and Care, Department of Neurology, David Geffen School of Medicine, University of California, Los Angeles, USA

**Keywords:** Neurodegenerative, Dementia, Neuropsychology, Cognitive impairment, Longitudinal, Neuropathology

## Abstract

Cerebrovascular disease (CVD) and Alzheimer’s disease (AD) often co-occur and may impact specific cognitive domains. This study’s goal was to determine effects of CVD and AD burden on cross-sectional and longitudinal executive function (EF) and memory in older adults.

Longitudinally followed participants from the National Alzheimer Coordinating Center database (n = 3342) were included. Cognitive outcomes were EF and memory composite scores. Baseline CVD presence was defined by moderate-to-severe white matter hyperintensities or lacunar infarct on MRI. Baseline AD pathology was defined by amyloid positivity via PET or CSF. Linear mixed models examined effects of CVD, AD, and time on cognitive outcomes, controlling for sex, education, baseline age, MoCA score, and total number of study visits.

At baseline, CVD associated with lower EF (p < 0.001), while AD associated with lower EF and memory (ps < 0.001). Longitudinally only AD associated with faster declines in memory and EF (ps < 0.001).

These results extend our understanding of CVD and AD pathology, highlighting that CVD does not necessarily indicate accelerated decline.

## 1. Introduction

Alzheimer’s Disease (AD) and cerebrovascular disease (CVD) are two of the most common etiologies for cognitive impairment in older adults. AD and CVD are often concurrent and can present similarly (Attems and Jellinger, 2014; Fernando and Ince, 2004; Jellinger and Attems, 2003). Gorelick et al. (2011) have also shown significant overlap in risk factors for CVD and AD, including hypercholesterolemia, hypertension, and diabetes mellitus. Clarifying the combined influences of CVD and AD pathologies on clinical presentation, however, is important for assessment and management of dementia patients. Literature is mixed on whether CVD and AD have synergistic or additive effects on cognitive impairment. Several longitudinal studies have found that amyloid pathology and white matter hyperintensity (WMH), a well-recognized biomarker for CVD (Wardlaw et al. 2013), may have independent and additive effects on cognitive impairment and future decline (Provenzano et al. 2013; Vemuri et al. 2015; Ye et al. 2015). Other studies, however, report synergistic effects of CVD and AD pathology on cognitive decline in individuals with early AD (Brickman et al. 2008; Gelber et al. 2012), with some evidence that CVD may promote the accumulation of AD tau pathology and accelerate AD-related neurodegeneration (Laing et al., 2020; Lao and Brickman, 2018).

The heterogeneity in these clinical findings may be partly related to the broad screeners often used to examine cognitive decline in large-scale datasets. In contrast, examining domain-specific longitudinal cognitive trajectories in patients with CVD and/or AD might help in further clarifying the combined roles of these pathologies on cognitive decline. CVD is hypothesized to contribute to worse executive functioning outcomes due to the common involvement of frontal white matter networks (e.g., fronto-striatal, fronto-parietal) in CVD (Mungas et al. 2001; Reed et al., 2007). However, while CVD associates with executive functioning outcomes in cross-sectional studies, these associations are not uniformly observed in longitudinal studies (Lao et al. 2022; Mungas et al. 2005). In contrast, AD pathology has demonstrated a consistent progressive cognitive profile across a wide variety of studies characterized primarily by declines in memory due to early involvement of medial temporal lobe structures (Baddeley et al. 1991; Jahn, 2013). Further large-scale evaluation of longitudinal cognitive profiles of CVD and AD would provide better insight into clinical presentation and contribute to improved differential diagnosis and subsequent clinical care and long-term planning.

The aims of this study were to use the National Alzheimer Coordinating Center (NACC) Uniform Data Set (UDS) to characterize cross-sectional and longitudinal associations of baseline biomarkers for CVD and AD with cognitive domains primarily affected in individuals with CVD and/or AD (e.g., executive function and episodic memory). Specifically, we examined the independent and interactive associations of these pathologies with cognitive functioning at baseline and on rates of decline over time. We hypothesized that: 1) CVD would be associated with worse executive function at baseline and over time, 2) AD pathology would be associated with worse memory at baseline and over time; and 3) CVD and AD would interact to synergistically relate to greater cognitive decline in both domains.

## 2. Methods

### 2.1. Participants

This study utilized data from the National Alzheimer’s Coordinating Center (NACC) database, which includes longitudinal clinical data from more than 42 Alzheimer’s Disease Research Centers (ADRCs) (Beekly et al. 2004, 2007). Standardized neuropsychological measures, demographic information, and medical history are collected annually. Parent study inclusion and exclusion criteria of participants vary according to each ADRC. Participants were included in the current study only if they had available biomarker data for CVD and AD pathology at baseline, resulting in a final sample of 3342 participants from the NACC database. This dataset includes NACC data from 2015 to 2021. All ADRCs obtained informed consent from all participants before each study visit as well as approval from institutional regulatory boards.

### 2.2. Measures

#### 2.2.1. Cognitive and Functional Assessment

Participants completed a comprehensive battery of cognitive tests from the Unified Data Set version 3 (UDS3) at each annual visit (Weintraub et al. 2018). Domain-specific composite scores were calculated from individual test scores using the following methods. To measure executive functioning, we utilized the validated UDS3-EF composite score (Staffaroni et al. 2021), which is calculated using item response theory and is comprised of Digit Span Backwards (total correct), Trail Making Tests parts A and B, lexical fluency (F and L words total correct), and semantic fluency (animal and vegetable fluency total correct). A memory composite z score was calculated by standardizing and averaging raw scores from UDS memory measures (i.e., Montreal Cognitive Assessment [MoCA] delayed recall score, Craft Story delayed recall [verbatim] score, and Benson figure recall score). Functional severity was assessed annually using the Clinical Dementia Rating (CDR), which is completed via an interview administered to participants’ study partners. The CDR assesses six cognitive and behavioral domains, including memory, orientation, judgment and problem solving, community activities, home and hobbies, and personal care. CDR scores of 0 indicate normal independent functioning, scores of 0.5 indicate mild difficulties within at least 1 domain, and scores of 1, 2, and 3 indicate dementia severity levels of mild, moderate, and severe, respectively.

#### 2.2.2. Cerebrovascular Disease (CVD)

All participants completed baseline magnetic resonance imaging (MRI) with a FLAIR sequence. MRI parameters and processing procedures have been described previously (Dadar et al., 2017). A full description of MRI methods can be found at https://www.alz.washington.edu/WEB/adni_proto.pdf. Baseline presence of CVD was determined by MRI evidence of lacunar infarct or moderate-to-severe WMH at the baseline visit (dichotomous yes/no). The Cardiovascular Health Study (CHS) score was used to quantify the extent of WMH (Manolio et al. 1994). A CHS score ranging from 5 to 8 corresponded with moderate-to-severe WMH.

#### 2.2.3. Alzheimer’s Disease (AD) Pathology

All participants completed either lumbar puncture or amyloid PET scan. Cerebrospinal fluid (CSF) was analyzed for Aβ_1–42_. PET imaging across ADCs used either PIB, Florbetapir, Florbetaben, or Flutemetamol as an amyloid tracer. Although different amyloid tracers were used across ADRC sites, each has been validated with sensitive and specific cut-points indicating elevated, pathological levels of cortical amyloid accumulation (Clark et al. 2011; Curtis et al. 2015; Price et al. 2005; Sabri et al. 2015). AD pathology in this sample was identified by a variable indicating the ADRC site’s determination regarding amyloid positivity (via amyloid PET or CSF AB) at the baseline visit (dichotomous yes/no). The choice to use amyloid positivity as evidence for AD pathology was based on established clinical guidelines for assessing biomarkers of AD pathological change (NIA-AA 2018). A subset of participants (N = 1018) also had tau measured at baseline via CSF (p-tau 181 reported value/concentration) or tau PET imaging with site-specific tracers used (University of Washington, 2015). Baseline tau positivity was identified via either of these methods at the local ADRC site (dichotomous yes/no).

#### 2.2.4. Cardiovascular risk

A subset of participants (n=1900) completed a clinical interview to collect information about current and past medical conditions, including hyperlipidemia, diabetes, and smoking history (current and past). Participants’ vitals were also taken annually, and hypertension was determined via validated systolic and diastolic blood pressure cutoffs of 140 and 80, respectively (Unger et al. 2020). A cardiovascular risk score was then calculated as a count of the presence of the following conditions: hypertension, hyperlipidemia, diabetes, and current smoking status [range = 0–4].

### 2.3. Statistical analysis

Linear mixed effects models were used to examine the associations of CVD and/or AD pathology with cognitive outcomes at baseline as well as with longitudinal cognitive trajectories. For each cognitive domain outcome (i.e., executive functioning and memory), we examined the full-factorial three-way interaction between time (years since baseline), CVD (yes/no), and AD (yes/no). If the three-way interaction was found to be non significant, it was removed from the models. Person-specific random intercepts and random effects of time were modeled, which allowed for examination of predictors of person-specific cognitive slopes. Covariates included baseline age, sex, years of education, their interactions with time, baseline MoCA score (to account for global cognition at baseline), and total number of study visits. Standardized regression estimates are reported, which represent the relationship between predictors and outcome in standard deviations units. R version 4.2.0 was used with the “lme4” package to conduct linear mixed effects regressions (Bates et al. 2015).

Based on findings from the primary analyses, several secondary analyses were conducted. First, to examine whether the observed relationships between neuropathology biomarkers and cognitive trajectories were preserved in participants for whom CVD or AD was detected in preclinical stages of disease, analyses were repeated in a subset of participants who were cognitively and functionally normal (i.e., CDR=0) at the baseline visit (n=1277). Next, given that cardiovascular risk factors are hypothesized to confer risk for cerebrovascular disease (Luchsinger and Mayeux, 2004; Santos et al. 2017), we conducted additional linear mixed effects models for each cognitive domain outcome examining the full-factorial three-way interaction between time, baseline AD, and baseline cardiovascular risk score. The same covariates and random effects from primary analyses were included in these models. Finally, to further characterize the influence of AD-related pathology, we conducted a “sensitivity” analysis in a subset of participants who had data on both amyloid and tau to examine the independent and interactive effects of CVD and tau positivity on cognitive trajectories.

## 3. Results

Baseline demographics by baseline biomarker status are displayed in Table 1. In the entire sample, participants were on average 70 years old, 52 % of participants were female, and 90 % of participants were White. Total number of visits ranged from 1 to 7, with participants remaining on study for on average 2 years. CVD−/AD− and CVD+/AD− participants were younger than CVD−/AD+ and CVD+/AD+ participants at baseline (p < 0.001). CVD+/AD+ participants had the lowest proportion of females (p < 0.001). CVD−/AD+ participants had the lowest proportion of White participants (p < 0.001). CVD−/AD− participants had the highest rates of being cognitively and functionally unimpaired (CDR = 0) at baseline (p < 0.001), as well as the greatest number of study visits and length of time in study (*p*s < 0.001). Of note, the range of total study visits was identical across all groups. 8 % of participants were missing data collected on individual imaging variables, but all had data available for the higher-level dichotomous CVD variable. Among the participants with CVD and individual imaging data, 31 % had lacunar infarcts, 64 % had moderate to extensive WMH, and 13 % had both lacunar infarcts and moderate to extensive WMH.

**Fig. 1.**
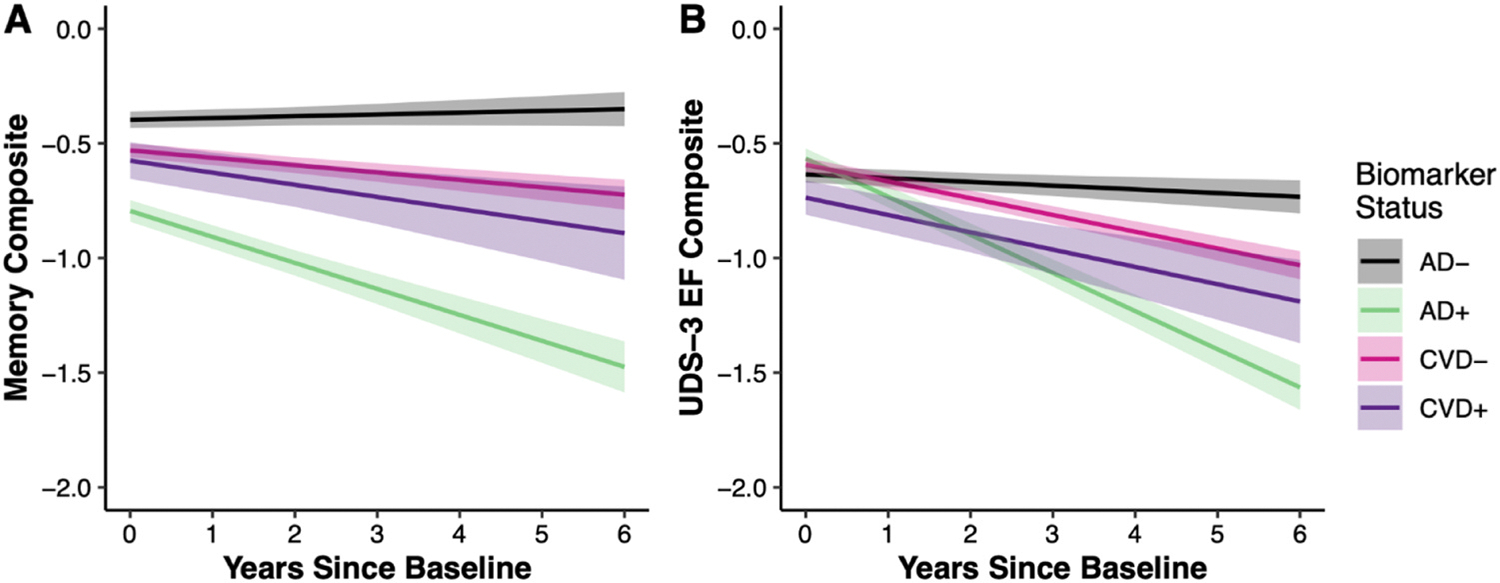
Estimated cognitive trajectories by baseline AD and CVD biomarker status for A) memory and B) executive functioning. 95 % confidence interval bands are included for each model.

Regarding longitudinal memory trajectories, the three-way interaction between CVD, AD, and time was not significant (*β* = 0.032, *p* = 0.535). Thus, the linear mixed effects model was rerun without the three-way interaction. Results of the final linear mixed effects model examining memory composite score trajectories are shown in Table 2. Conditional main effects of CVD and AD indicate that CVD did not strongly relate to baseline memory scores (*β* = −0.033, *p* = 0.579), whereas AD was significantly associated with lower baseline memory scores (*β* = −0.566, *p* < 0.001). The interaction between CVD and AD on baseline memory was not significant (*β* = −0.061, p = 0.463). Interactions between each biomarker and time, however, showed that baseline presence of AD related to significant declines in memory over time (*β* = −0.178, *p* < 0.001), while the association between baseline CVD and memory decline did not reach statistical significance (*β* = −0.031, *p* = 0.248; Fig. 1A). Taken together, non-significant interactions between CVD and AD suggest that these pathologies did not have a synergistic effect on baseline memory nor rate of memory change.

Similarly, the three-way interaction between CVD, AD, and time on executive functioning trajectories was not significant (*β* = 0.041, *p* = 0.419). Thus, the linear mixed effects model was rerun after removing the three-way interaction. Results of the final linear mixed effects model examining executive function composite score trajectories are shown in Table 3. Conditional main effects demonstrate that both CVD (*β* = −0.146, *p* < 0.001) and AD significantly related to lower baseline executive function composite scores (*β* = −0.165, *p* < 0.001; Fig. 1B). The interaction between CVD and AD on baseline executive function was not significant (*β* = 0.115, p = 0.131). Baseline AD related to significant declines in executive function over time (*β* = −0.243, *p* < 0.001); however, the association between baseline CVD and executive function decline over time did not reach statistical significance (*β* = −0.004, *p* = 0.88). Again, these non-significant interactions between CVD and AD suggest that these pathologies did not have a synergistic effect on baseline executive functioning nor rate of executive functioning change.

Secondary analyses examining only the 1277 participants who were cognitively and functionally normal at baseline (CVD−/AD−: n=963; CVD+/AD−: n=54;CVD−/AD+: n=240;CVD+/AD+: n=20) showed that the pattern of results was consistent in this subset. Briefly, the three-way interaction was not significant for either cognitive outcome (*p*s > 0.297) and was removed from subsequent analyses. Baseline AD continued to show the largest effects on cognitive decline for both memory (*β* = −0.12, p < 0.001) and executive functioning (*β* = −0.14, p < 0.001), while baseline presence of CVD was not strongly associated with cognitive decline in either domain (*p* = 0.64 for memory, p = 0.94 for executive function). Baseline CVD continued to be significantly associated with baseline executive functioning performance (*β* = −0.26, *p* < 0.001), but not with baseline memory (*β* = −0.001, *p* > 0.99). Baseline AD was not associated with baseline performance in executive functioning (*p* = 0.02), but was significantly associated with baseline performance in memory (*β* = −0.18, *p* < 0.001).

Additional secondary analyses also examined cardiovascular risk in the subset of 1900 participants with available cardiovascular risk factor data, given the well-established relationship between cardiovascular risk factors and CVD. 12 % of participants were reported to have diabetes at baseline and 50 % of participants were reported to have hypercholesterolemia at baseline, as assessed by a clinician upon initial visit. 3 % of participants reported having smoked within the past 30 days, and 35 % of participants had hypertension based off validated systolic and diastolic cutoffs. Participants with CVD had expectedly higher baseline cardiovascular risk scores (mean = 1.41, SD = 0.91) than those without CVD (mean = 1.14, SD = 0.85; t_370_ = −4.62, p < 0.001). Linear mixed effects models assessing the relationships between cardiovascular risk, AD, and cognitive trajectories showed a very similar pattern of results as the primary analyses. Specifically, cardiovascular risk score was associated with lower executive function at baseline (*β* = −0.210, *p* < 0.001) but not longitudinal executive function trajectories (*p* = 0.225). Cardiovascular risk score was also not significantly related to memory at baseline (*p* = 0.661) nor longitudinally (*p* = 0.627). Cardiovascular risk also did not significantly interact with AD on longitudinal memory (*p* = 0.496) nor executive function outcomes (*p* = 0.119).

In the subset of 1018 participants with data on both amyloid and tau, 12 participants who were tau positive and amyloid negative were excluded to ensure capture of AD-related pathology only. Models examining the independent and interactive effects of CVD and tau positivity on cognitive trajectories in this subset showed the same pattern of results as that from the primary analyses that defined AD solely based on amyloid positivity, such that the 3-way interactions between CVD, tau, and time were not statistically significant for memory or executive functioning. Taken together with the primary analysis, these results highlight the importance of characterizing *any* AD pathologic change, including amyloidosis alone.

## 4. Discussion

Improving our understanding of the interplay between CVD and AD on clinical presentation and progression, as well as specific cognitive domain involvement, is relevant given the context of high rates of comorbid neuropathologies and mixed findings in previous studies. Consistent with widely held clinical assumptions and the broader literature, our results showed that at baseline, neuroimaging evidence of CVD related to worse executive function, while AD biomarker positivity related to worse performance in both memory and executive function domains. Inconsistent with our hypothesis that CVD and AD would synergistically exacerbate cognitive decline, our longitudinal model showed that AD pathology was the primary driver of decline in both executive function and memory regardless of baseline CVD presence. Taken together, findings suggest that although CVD relates to an initial hit to executive functioning, CVD-related cognitive decline is not necessarily progressive and CVD may not modify rates of cognitive decline when comorbid with AD.

The results from our three-way full-factorial model showing a lack of synergy between CVD and AD on cognitive decline align with the findings of some recent studies (Vemuri and Knopman, 2016), but are inconsistent with others demonstrating synergistic effects between these pathologies (Brickman et al. 2008). Discrepancies across studies may be partially explained by methods used to define CVD. Many studies including ours define presence of WMH on neuroimaging as evidence of CVD pathology, as WMH have been consistently found in the context of stroke and intracranial atherosclerosis, and is increased in those with cardiovascular risk factors (e.g., hypertension, diabetes, smoking) (Pålhaugen et al. 2021); however, WMH may also reflect more heterogeneous sources of white matter injury. Recent studies have demonstrated that some WMH, particularly in posterior brain regions, may be more related to AD pathology than to vascular disease (Gaubert et al. 2021; McAleese et al., 2017). Further examination of regional specificity of associations between WMH and cognition in participants with AD and CVD pathology in several recent studies has suggested a predominance of WMH distribution in posterior regions correlating with heavy amyloid burden (Garnier-Crussard et al. 2022; Gaubert et al. 2021) and predominance of WMH in anterior regions association with greater CVD risk (Brickman, 2013; Pålhaugen et al. 2021). Our study did not examine regional distributions of WMH; rather, we categorized participants based on presence of any moderate-to-severe white matter injury. However, our posthoc analysis involving cardiovascular risk factors paralleled the CVD models, which bolsters support for our primary analyses suggesting that WMH in our analyses indeed reflect CVD processes.

Regarding our cognitive domain-specific findings, CVD has frequently been shown to associate with executive dysfunction. Lacunar infarcts and white matter lesions, which are known to be major pathological features of CVD, have been observed to occur predominantly in the frontal lobe when representative of CVD. Lesions in the frontal cortex may lead to declines in executive function due to interruption of frontal white matter circuits (Tullberg et al. 2004). This is consistent with our finding of the relationship between CVD and worse executive function at baseline. However, we did not observe an association between CVD and executive function longitudinally. We currently do not have data on longitudinal WMH progression, but future studies should examine this aspect.

AD, on the other hand, is known to be progressive. Our findings are highly consistent with the vast literature showing how AD pathology targets medial temporal lobe structures important for memory early in the disease course, then spreads globally (Arnold et al. 1991; Jahn, 2013; Lace et al. 2009). This AD-related progression is evidenced in our primary results showing AD pathology’s association with both baseline memory and executive functioning, as well as progressive declines in both domains. In addition to the very well-established relationship between AD and memory, our observed baseline and longitudinal relationships between AD pathology and executive functioning are also supported by other previous studies which have demonstrated statistically significant declines in longitudinal executive functioning in participants with AD pathology as well as loss of executive functioning at baseline (Staffaroni et al. 2021).

Importantly, all of our findings held when examining only the subset of participants who were cognitively and functionally normal at baseline. This supports that our findings were not being driven by participants with more severe baseline clinical presentations. This result also has important clinical implications that identifying pathological markers of CVD and AD are predictive of long-term clinical outcomes even in those without any symptoms. Our sensitivity analysis additionally bolsters support for quantification of any AD-related biomarker, even amyloidosis alone, as the inclusion of AD-tau data in our categorization of baseline AD presence did not alter the observed pattern of results. It should also be noted that baseline executive functioning was lower in the CVD+/AD+ group than that of the CVD−/AD− group. Although we did not observe synergistic longitudinal effects of CVD and AD on cognitive decline, this lower baseline level of executive functioning may still represent a pathway to faster conversion from mild cognitive impairment to dementia.

Some strengths of this study include a large sample size, as well as use of composites for cognitive outcomes; however, there are limitations that must be addressed. First, the pathology data used in this study was limited in a number of ways. Although treating AD pathology and WMH as dichotomous variables is clinically relevant through the use of established thresholds, it limited our ability to examine the full range of pathological burden for each condition. In addition, we were unable to study more nuanced aspects of CVD pathology, including shape or regional distributions of lacunar infarcts and WMH, as these raw imaging features and uniform CHS scoring across participants were not available. This significantly limited our ability to conduct focused analyses. Another limitation is a lack of longitudinal information about the progression of AD and CVD pathology and the higher rates of attrition in the biomarker positive groups, which limited our ability to understand the temporal tracking between pathology burden and cognitive manifestation. Additionally, there is a significant lack of diversity in the NACC cohort, which limits the generalizability of our findings to demographically underrepresented older adults. Despite these limitations, the strong relationships observed highlight the robustness of our findings.

### 4.1. Conclusions

In summary, this study builds on our understanding of how CVD and AD pathologies impact cognitive aging. Our findings have implications for longitudinal studies of cerebrovascular disease, and suggests the important of analyzing presence of AD biomarkers alongside WMH. Our results indicate the significance of AD pathology in not only progressive memory loss but also in executive function declines over time, regardless of CVD presence. Furthermore, when taken alongside the findings from our cardiovascular risk factors model, our CVD-related findings suggest that presence of CVD along with AD pathology is not necessarily indicative of accelerated cognitive decline. These encouraging results highlight the potential for cognitive stability in those without AD even after CVD-related white matter injuries have occurred, while also emphasizing the need to better understand contributions of more specific types of WMH that may confer greater or less risk for cognitive decline.

## Figures and Tables

**Table 1:** Demographic and clinical characteristics (N = 3342).

	Baseline Biomarker Positivity					

	A. Neither CVD nor AD	B. CVD only	C. AD only	D. CVD & AD	P	Pairwise

N	1701	216	1211	214		

Baseline Age	68.99 (8.86)	74.26 (8.16)	69.43 (8.70)	75.45 (7.55)	<0.001	A,C < B,D
Education	16.76 (6.88)	17.09 (10.13)	16.76 (7.62)	16.42 (6.29)	0.824	
Sex (female)	913 (54 %)	110 (51 %)	632 (52 %)	91 (43 %)	0.022	A,B,C > D
Race/Ethnicity (White)	1488 (88 %)	181 (84 %)	1135 (94 %)	196 (92 %)	<0.001	B < C,D
Baseline CDR					<0.001	
0 (normal)	963 (57 %)	54 (25 %)	240 (20 %)	20 (9 %)		A > B,C > D
0.5 (mild)	564 (33 %)	126 (58%)	640 (53 %)	118 (55 %)		A < B,C,D
1+ (dementia)	174 (10 %)	36 (17 %)	331 (27 %)	76 (36 %)		A < B < C < D
History of stroke	0 (0 %)	26 (12 %)	0 (0 %)	11 (5 %)	<0.001	A,C < D < B
Total years on study	2.44 (1.94) [range: 0–6.4]	1.87 (1.62) [range: 0–6.2]	1.73 (1.66) [range: 0–6.5]	1.56 (1.57) [range: 0–6.3]	<0.001	A > B,C,D
Total number of visits	3.05 (1.69) [range: 1–7]	2.60 (1.45) [range: 1–7]	2.46 (1.46) [range: 1–7]	2.36 (1.39) [range: 1–7]	<0.001	A > B,C,D

Note. Values are mean (SD) or n (%). Pairwise comparisons were conducted using Tukey’s HSD.

**Table 2:** Results of linear mixed effects model examining baseline presence of CVD and/ or AD as predictors of memory trajectories, covarying for age, education, sex, number of visits, and baseline MoCA score.

Predictor	β	95 % CI	P-value

Time	0.022	−0.002, 0.046	0.074
CVD (ref: CVD−)^a^	−0.033	−0.151, 0.085	0.579
AD (ref: AD−)^a^	−0.566	−0.631, −0.501	<0.001
CVD * AD^a^	−0.061	−0.224, 0.102	0.463
CVD * Time	−0.031	−0.082, 0.021	0.248
AD * Time	−0.178	−0.211, −0.146	<0.001
Baseline age^a^	−0.115	−0.143, −0.087	<0.001
Baseline MoCA^a^	0.744	0.717, 0.77	<0.001
Education (yrs)^a^	−0.017	−0.044, 0.009	0.192
Sex (ref: men)^a^	0.132	0.075, 0.188	<0.001
Number of Visits	0.061	0.031, 0.09	<0.001
Age * Time	−0.04	−0.055, −0.024	<0.001
Education * Time	−0.002	−0.019, 0.016	0.863
Sex * Time	−0.009	−0.038, 0.02	0.541

Notes. CVD = cerebrovascular disease; AD = Alzheimer’s disease; MoCA = Montreal Cognitive Assessment. CVD- = no baseline presence of CVD; AD- = no baseline presence of AD biomarkers.

a Effect at baseline (outcome at time = 0)

**Table 3:** Results of linear mixed effects model examining baseline presence of CVD and/ or AD as predictors of executive function trajectories, covarying for age, education, sex, number of visits, and baseline MoCA score.

Predictor	β	95 % CI	P-value

Time	−0.048	−0.074, −0.023	<0.001
CVD (ref: CVD−)^a^	−0.146	−0.232, −0.061	<0.001
AD (ref: AD−)^a^	−0.165	−0.223, −0.106	<0.001
CVD * AD^a^	0.115	−0.034, 0.265	0.131
CVD * Time	−0.004	−0.055, 0.048	0.880
AD * Time	−0.243	−0.275, −0.211	<0.001
Baseline Age^a^	−0.078	−0.105, −0.051	<0.001
Baseline MoCA^a^	0.877	0.852, 0.902	<0.001
Education^a^	0.026	0.001, 0.051	0.042
Sex (ref: men)^a^	0.159	0.104, 0.213	<0.001
Number of Visits	0.108	0.08, 0.137	<0.001
Age * Time	−0.003	−0.019, 0.012	0.671
Education * Time	0.003	−0.015, 0.021	0.734
Sex * Time	0.049	0.019, 0.08	0.002

Notes. CVD = cerebrovascular disease; AD = Alzheimer’s disease; MoCA = Montreal Cognitive Assessment. CVD- = no baseline presence of CVD; AD- = no baseline presence of AD biomarkers.

a Effect at baseline (outcome at time = 0)
